# Characterizing the NLRP3 Inflammasome in Mood Disorders: Overview, Technical Development, and Measures of Peripheral Activation in Adolescent Patients

**DOI:** 10.3390/ijms222212513

**Published:** 2021-11-19

**Authors:** Xinyang Zhou, Shehan M. Fernando, Alexander Y. Pan, Rebecca Laposa, Kathryn R. Cullen, Bonnie Klimes-Dougan, Ana C. Andreazza

**Affiliations:** 1 Department of Pharmacology, University of Toronto, Toronto, ON M5S 1A8, Canada; xinyang.zhou@mail.utoronto.ca (X.Z.); mark.fernando@mail.utoronto.ca (S.M.F.); alex.pan@utoronto.ca (A.Y.P.); rebecca.laposa@utoronto.ca (R.L.); 2 Department of Psychiatry and Behavioral Sciences, University of Minnesota, Minneapolis, MN 55454, USA; rega0026@umn.edu; 3 Department of Psychology, University of Minnesota, Minneapolis, MN 55455, USA; klimes@umn.edu; 4 Department of Psychiatry, University of Toronto, Toronto, ON M5S 1A8, Canada

**Keywords:** NLRP3, ASC specks, IL-1 beta, bipolar, depression, inflammation

## Abstract

The NOD-, LRR-, and pyrin-domain-containing protein 3 (NLRP3) inflammasome is a node of intracellular stress pathways and a druggable target which integrates mitochondrial stress and inflammatory cascades. While a body of evidence suggests the involvement of the NLRP3 inflammasome in numerous diseases, a lack of reliable measurement techniques highlights the need for a robust assay using small quantities of biological samples. We present a literature overview on peripheral activation of the NLRP3 inflammasome in mood disorders, then outline a process to develop and validate a robust assay to measure baseline and activated intracellular levels of “apoptosis-associated speck-like protein containing a CARD” (ASC) as a key component of an inflammatory profile in peripheral blood mononuclear cells (PBMC). A consistent association between high NLRP3 mRNA levels and relevant cytokines was seen in the literature. Using our method to measure ASC, stimulation of PBMC with lipopolysaccharide and nigericin or adenosine triphosphate resulted in microscopic identification of intracellular ASC specks, as well as interleukin 1 (IL-1) beta and caspase-1 p10 in the periphery. This was abolished by dose-dependent pre-treatment with 100 nM MCC950. We also report the use of this technique in a small pilot sample from patients with bipolar disorder and depressive disorders. The results show that levels of intracellular ASC and IL-1 beta are sensitive to change upon activation and maintained over time, which may be used to improve the detection of NLRP3 activation and guide personalized therapeutic strategy in the treatment of patients.

## 1. Introduction

Inflammasomes are multiprotein complexes which are activated via infectious microbes and other molecules derived from host proteins [1]. The inflammasomes regulate the activation of caspase-1 and induce inflammation [2]. An example is the nucleotide binding domain (NOD)-, leucine-rich repeat (LRR)-, and pyrin domain (PYD)-containing protein 3 (NLRP3) [3]. There are significant differences in how each individual inflammasome complex assembles after recognition of its specific stimuli [4]. In NLRP3, this occurs through its pyrin domain, which associates with a protein known as apoptosis-associated speck-like protein containing a CARD (ASC) [5]. Procaspase-1 is believed to be activated by dimerization, and through autoproteolytic processing, activates important pro-inflammatory cytokines including interleukin 1 (IL-1) beta and IL-18 [6].

The NLRP3 inflammasome is activated through a two-step activation process [7,8]. The system first needs to be primed through activation of nuclear factor kappa-B (NF-κB) to upregulate the expression of NLRP3 [9]. This can be achieved in a variety of ways, including lipopolysaccharide (LPS) binding to a receptor on the surface of innate immune cell, or through induction of a NOD2 cytosolic pattern recognition receptor [10,11,12]. Following post-translational modification of ASC, such as ubiquitination or phosphorylation, the inflammasome is assembled and NLRP3 can be activated by a large array of stimuli, including mitochondrial DNA, ATP, reactive oxygen species, and foreign pathogens [13,14].

Activation of the NLRP3 inflammasome leads to the release of pro-inflammatory cytokines (IL-1 beta and IL-18) and reactive oxygen species from microglia [15,16]. These mediators cause damage to myelin and induce pathological modifications to astrocytes and neurons, which may potentially alter neuronal growth and communications and contribute to the progression of mood disorders and neuropsychiatric conditions [17].

Pharmacological modulation of the NLRP3 inflammasome through inhibition holds promise in the management of inflammatory diseases [18]. Several major neuropsychiatric illnesses, including mood disorders, exhibit both mitochondrial dysfunction and elevated inflammation; yet treating either separately has not been clinically effective [19]. The NLRP3 inflammasome integrates mitochondrial stress and triggers inflammatory responses upon activation [14]. MCC950 is a well-known inhibitor which prevents the assembly of NLRP3, and crucially holds clinical promise for therapeutic applications [20]. Other inhibitors of NLRP3 are also available, and operate through a variety of mechanisms, including direct inhibition (OLT1177, methylenedioxy-beta-nitrotyrosine, tranilast), indirect inhibition (glyburide, 16673-34-0, FC-11A2, beta-hydroxybutyrate), or both (parthenolide, BAY 11-7082) [21].

In this study we (1) present an overview of the literature on peripheral (i.e., human peripheral blood mononuclear cells (PBMC)) activation of the NLRP3 inflammasome in mood disorders; (2) demonstrate the development and validation of an appropriate technical approach using two concurrent detection methods: immunofluorescence and protein levels to study the activation of the NLRP3 system in PBMC; (3) apply this assay in a pilot clinical sample set of adolescent patients with mood disorders, i.e., bipolar disorder (BD) or depressive disorder (DD).

One of the major problems in the literature is the lack of a well described technique that assesses the activation of NLRP3 in peripheral samples. The vast majority of studies report an increase on end products (IL-1 beta or caspase-1) or assess the NLRP3 via mRNA or protein levels, which do not provide direct evidence of activation of the NLRP3 system and its sensitivity to stimuli [1,22,23]. Measuring activation of the NLRP3 system itself can help to define the source of inflammation and guide therapeutic strategy.

## 2. Results

### 2.1. Overview Demonstrates the Relevance of NLRP3 Inflammasome in Mood Disorders

To date, we identified seven articles investigated NLRP3 inflammasome activation in neuropsychiatric patients suffering from mood disorders, including major depressive disorder (MDD) and bipolar disorder [24,25,26,27,28,29,30]. Across studies investigating these mood disorders, the techniques used to measure the NLRP3 inflammasome were real-time quantitative reverse transcription polymerase chain reaction (qRT-PCR), enzyme linked immunosorbent (ELISA), and Western blotting (Table 1).

Overall, the studies report an increase in mRNA expression of NLRP3, ASC, and caspase-1 genes in mood disorder patient groups when compared to healthy controls. This is confirmed with an increase in protein levels of IL-1 beta and/or IL-18 cytokine production in the corresponding blood samples. Of note is the 2017 study by Alcocer-Gómez et al., who demonstrated decreased levels of the NLRP3 inflammasome in MDD patients taking medication. No studies measured ASC protein levels (Table 2, outlined in dark red) and no endpoints were measured using techniques other than traditional assays for mRNA and protein expression. Given that alterations in mRNA expression do not necessarily translate to changes in protein expression, results showing increases in both mRNA and protein expression provide broader insight when assessing inflammasome activation.

### 2.2. A Simple Immunofluorescence Assay to Measure ASC Speck in PBMC—A Way Forward to Characterize NLRP3 Activation in Disease

**Figure 1:**
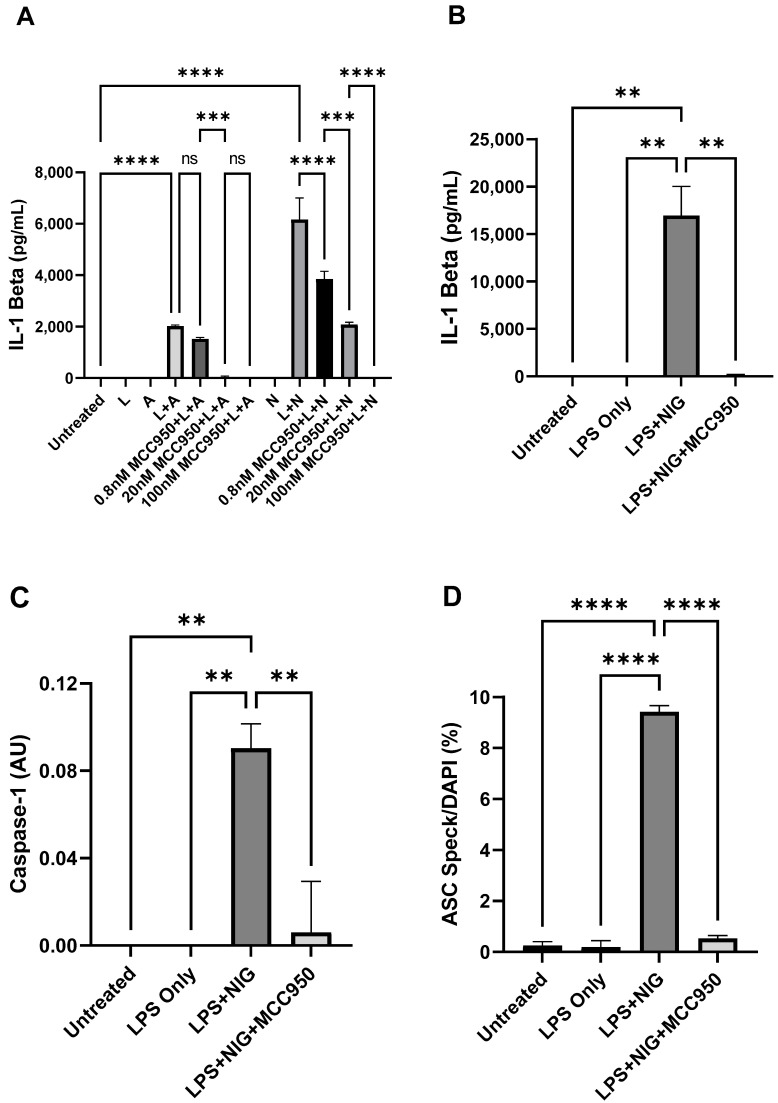
Validation of NLRP3 inflammasome endpoints in healthy PBMC. (**A**) Response of IL-1 beta with increasing doses of NLRP3 inhibitor MCC950 prior to stimulation with 100 ng/mL LPS and 10 µM nigericin or 5 mM ATP in 1 million cells per mL, measured by ELISA. (**B**,**C**) Levels of IL-1 beta (**B**) and Caspase-1 (**C**) in cell culture supernatants under NLRP3 activator and inhibitor conditions in 2 million cells per mL, as measured by ELISA; 100 nM of MCC950 was used in inhibitor treatments. AU refers to arbitrary units. (**D**) ASC Speck formation under various conditions as measured by fluorescence microscopy. Data in (**A**) are expressed as the mean and standard deviations of two technical replicates from a single experiment, while data in (**B**–**D**) are expressed as the mean and standard deviations of two independent experiments. One way ANOVA followed by Šídák’s multiple comparison test was used to assess differences between groups (** adj. *p* < 0.01, *** adj. *p* < 0.001, **** adj. *p* < 0.0001).

Stimulation of PBMC with LPS followed by 10 µM nigericin (NIG) or 5 mM adenosine triphosphate (ATP) induced an elevation in secreted IL-1 beta levels, which were inhibited by MCC950 (0.8 nM, 20 nM, 100 nM) in a dose-dependent manner (F = 115.8, *p* < 0.0001, Figure 1A). Post-hoc analysis revealed significant differences between dose–response treatments of MCC950 with nigericin (*** adj. *p* < 0.01, **** adj. *p* < 0.0001), but not between all MCC950 treatments for ATP (Figure 1A).

MCC950 was chosen due to its potency and ability to specifically inhibit NLRP3 inflammasome activation, block caspase-1 activation, and reduce IL-1 beta processing and secretion. Together, these results demonstrate that the cell culture and treatment process are producing a robust activation of NLRP3 for nigericin. Administration of LPS, ATP, or nigericin alone does not induce a response in IL-1 beta levels, which supports literature consensus that NLRP3 activation requires both a priming agent and activator to detect changes in endpoints (Figure 1B–D).

**Figure 2:**
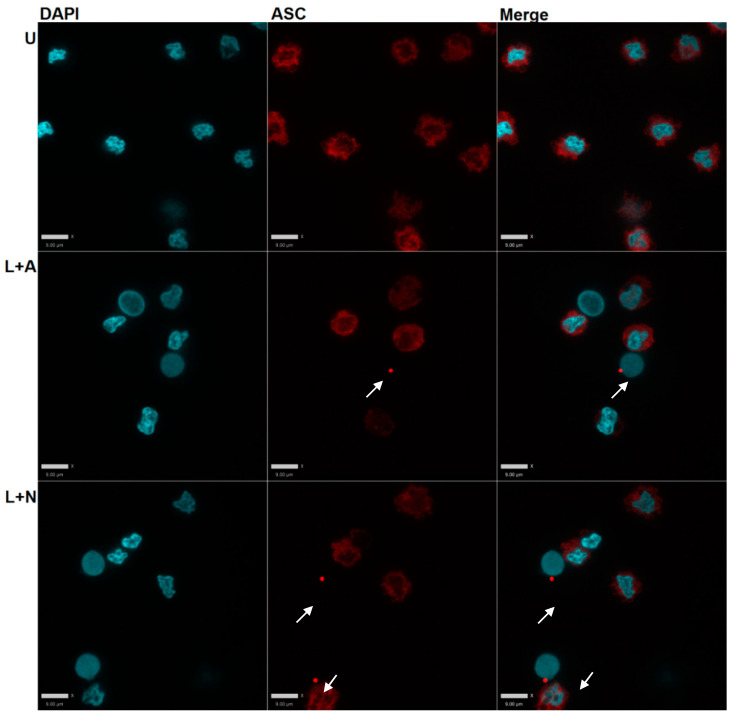
Photomicrographs of PBMC with ASC specks (shown with arrows). Visible ASC specks form when PBMC are treated with NLRP3 inflammasome activators. Scale bar, images were not zoomed or cropped = 9 μm. U—unstimulated; L—LPS; A—ATP; N—Nigericin. Images captured at 63X objective lens magnification.

Stimulation of PBMC with LPS and nigericin or ATP resulted in the formation of ASC foci in some cells (i.e., ASC ‘specks’) (Figure 2), which were not present in the absence of a specific anti-ASC antibody. We observed poorer cell attachment in cells treated with LPS + ATP compared to LPS + nigericin. Nigericin also elicited more robust IL-1 beta responses than ATP (Figure 2); for these reasons, nigericin was used in subsequent experiments.

Extracellular IL-1 beta secretion, caspase-1 p10, and intracellular ASC speck formation were further assessed in additional experiments. Stimulation with LPS and nigericin induced marked responses in IL-1 beta, caspase-1 p10, and ASC speck formation, which were abolished by pre-treatment with 100 nM MCC950 (Figure 1B–D). The simultaneous measurement of ASC specks, caspase-1, and IL-1 beta compared to MCC950 inhibitor presence provided three-fold confirmation of the assay’s ability to measure consistent directional change in NLRP3 inflammasome endpoints.

These results suggest that the endpoints adequately measure NLRP3 activation, though we observed the most robust responses with IL-1 beta, which frequently exceeded the upper limit of detection without dilution. As such, IL-1 beta and ASC speck formation can be selected for measurement in human patient samples to assess NLRP3 activation.

### 2.3. Assessment of NLRP3 Inflammasome Activation in Adolescent Patients with Mood Disorders

Seven adolescents with diagnosis of mood disorder (bipolar spectrum disorders or a depressive disorder), aged 13 to 19, were selected from a larger study of mixed diagnosis (Table 3). This specific subsample was selected due to the availability of NLRP3 endpoint measures at both recruitment (T1) and after 6 months (T2) to verify the ability of the assay to consistently demonstrate ASC and IL-1 beta activation over time. Whole blood samples were collected for further extraction of plasma (measurement of IL-1 beta) and PBMC (measurement of ASC specks).

#### 2.3.1. Activation of NLRP3 Inflammasome in Untreated PBMC Versus Treated PBMC

**Figure 3:**
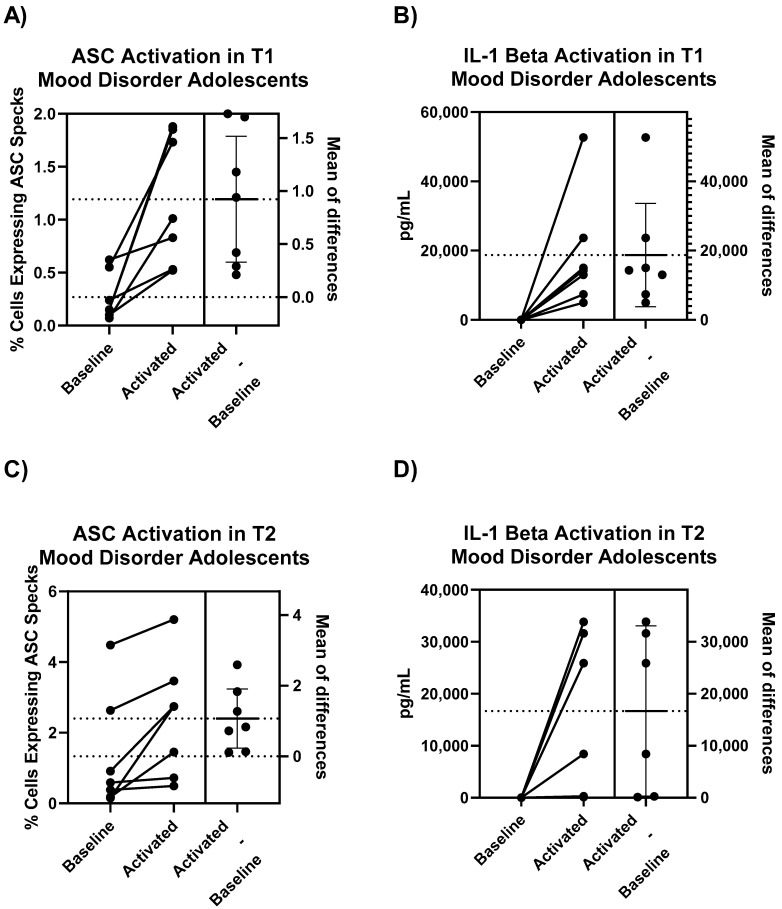
Paired *t*-test results demonstrating activation of (**A**) ASC and (**B**) IL-1 beta in untreated PBMC of adolescent mood disorder patients compared to LPS and nigericin activated PBMC from the same patient. Results from the T2 visit 6 months later for (**C**) ASC and (**D**) IL-1 beta were also measured. ASC values are intracellular and measured using immunofluorescence while IL-1 beta values are extracellular and measured using ELISA. Mean of differences are expressed as mean ± SD, all paired *t*-tests were statistically significant for *p* < 0.05.

Intracellular ASC specks, presented as percentage of cells expressing ASC specks, in untreated PBMC (baseline) compared to LPS and nigericin treated PBMC (activated) showed a significant difference (t = 3.801, mean of differences ± standard deviation (SD) = 0.9243 ± 0.6433%, *p* = 0.0090, Figure 3A), which was further confirmed with levels of extracellular IL-1 beta production from the same corresponding patient samples (t = 3.071, mean of differences = 18,718 ± 16126pg/mL, *p* = 0.0219, Figure 3B). This was also observed for samples assayed from the second follow-up visit (T2) conducted 6 months later for both ASC (t = 3.124, mean of differences = 1.069 ± 0.9049, *p* = 0.0205, Figure 3C) and IL-1 beta (t = 2.622, mean of differences = 16,680 ± 15582pg/mL, *p* = 0.0470, Figure 3D).

#### 2.3.2. Comparison of NLRP3 Inflammasome in Adolescent Mood Disorder Patients over Time

**Figure 4:**
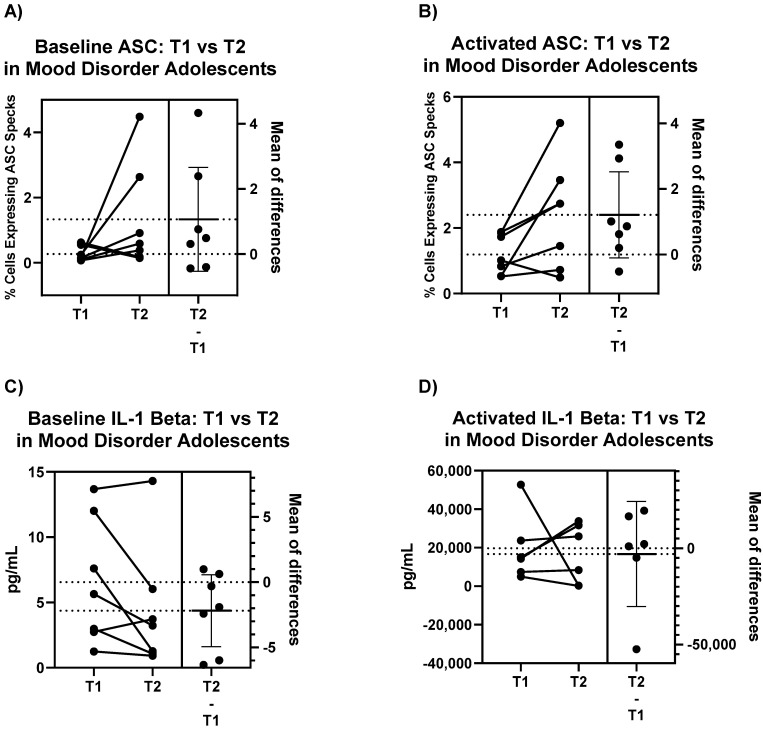
Paired *t*-test results comparing change in ASC specks in (**A**) untreated and (**B**) treated PBMC of adolescent mood disorder patients from first visit to second visit 6 months later, with corresponding change in IL-1 beta for (**C**) untreated and (**D**) treated PBMC. ASC values are intracellular and measured using immunofluorescence while IL-1 beta values are extracellular and measured using ELISA. Mean of differences are expressed as mean ± SD, all paired *t*-tests were not statistically significant for *p* < 0.05.

The differences in ASC specks are higher in samples from the second visit (T2), both for baseline PBMC measurements and for activated PBMC (Figure 4A,B), while some patients show a decrease in IL-1 beta expression (Figure 4C,D). However, these results were not statistically significant (A) *p* = 0.1539, (B) *p* = 0.0652, (C) *p* = 0.0990, (D) *p* = 0.7893. Interestingly, the mean of differences for ASC specks is approximately consistent for both baseline and activated PBMC in ASC (Figure 4A,B) and IL-1 beta (Figure 4C,D).

## 3. Discussion

We are increasingly understanding the role of inflammation in psychiatric disease [23,31,32,33,34,35,36]. As evidenced by our literature overview, there is evidence to support upregulation of the NLRP3 inflammasome in mood disorders [24,25,26,27,28,29,30]. However, a lack of reliable measurement techniques in psychiatric conditions highlight the need for a robust assay, which may be used as a component of disease diagnosis and drug screening.

To date, many studies arrive at conclusions about the NLRP3 inflammasome based entirely on measurements of gene expression and variable techniques, such as Western blot without measurement of ASC protein to capture differences [24,25,26,27,28,29,30]. We argue that this alone is not a sufficient measure of NLRP3 activation. We developed and validated an assay to measure intracellular formation of ASC specks via immunofluorescence. Our assay reliably assesses the levels of NLRP3 inflammasome activation in PBMC. Given the challenge of detecting biomarkers in quantity limited biological samples, there is need for an appropriate technical approach to study and measure proteins at a higher sensitivity within tissue.

Fluorescence microscopy of intact PBMC with antibody-based detection of endogenous ASC protein is a promising approach due to its physiological relevance to the immune system [11]. ASC aggregate into small yet densely packed specks that can be robustly quantified using size and fluorescence intensity thresholds [37,38]. We have outlined a process for assessing NLRP3 inflammasome activation, applied the technique in PBMC from adolescents with mood disorders, and validated the assay with measurements in cell culture media of corresponding samples.

The results of our experiment should be taken with caution due to the small sample size. Some patients are taking medications, which may affect levels of ASC and IL-1 beta. It is worthwhile to note that antidepressants have been shown to modulate IL-1 beta levels [26], therefore it is imperative to further investigate NLPR3 inflammasome activation in a larger sample size which properly controls for the effects of medication. However, even with these limitations, we observed some subtle differences between individual adolescents, which has previously not been well explored.

In early-stage mood disorder patients, we see that all PBMC are sensitive to NLRP3 activation, as evidenced by the significant increase in intracellular ASC specks and extracellular IL-1 beta production (Figure 3). This activation response, in both baseline and treated samples, is retained in PBMC samples extracted 6 months later, as suggested by the lack of significant differences across T1 and T2 timepoints (Figure 4). It is important to note that while all patients demonstrate significant increases in intracellular ASC specks and extracellular IL-1 beta production upon activation, there is variability in the sensitivity to activation, which has also been previously demonstrated for cytokine release [39]. The inter-individual differences in activation magnitude may inform individualized therapeutic treatments to control the level of NLRP3 activation in patients with mood disorders and other related diseases.

This research has important clinical implications. From a strategic standpoint, NLRP3 is a druggable target with available inhibitors [20,21]. There are no active clinical trials for patients with mood disorders using NLRP3 inhibitors, nor any psychiatric-purpose patents for NLRP3 inhibitors. We propose the NLRP3 pathway as a potential drug target and biological endpoint, and NLRP3 inhibitors as candidates for early intervention and combination therapy. Our conceptual framework incorporates a two-hit model for NLRP3 inflammasome activation, wherein the combination of both mitochondrial reactive oxygen species and inflammatory stimuli are required for activation of the NLRP3 inflammasome [14,17,19,29]. We envision that in clinical practice, with the use of an appropriate assay that simultaneously detects activated ASC protein and IL-1 beta, peripheral NLRP3 activation can be tracked to monitor therapeutic efficacy.

In conclusion, we have combined measurements involving immunostaining, microscopy, and protein biochemistry to create a characterization for representing NLRP3 inflammasome activation. This assay has the potential to help physicians by providing a biological indicator using quantity-limited clinical specimens to facilitate disease management.

## 4. Materials and Methods

### 4.1. Overview of Evidence on Peripheral Activation of the NLRP3 Inflammasome

An overview of the literature investigated inflammasome activation in human peripheral blood mononuclear cells (PBMC). The search strategy covered three major overlapping areas: articles assessing inflammasomes (1) levels of activity; (2) measured in peripheral blood leukocytes; (3) from human patients. Relevant articles from biomedical literature were retrieved from the Medline and Embase databases using the OVID search tool, using three Boolean “AND” operators as intersects to retrieve articles fulfilling these major requirements. Throughout the screening process, select articles [24,26] were used as controls to verify the accuracy and effectiveness of each search strategy iteration. This approach returned 7 articles involving patients with diagnosis of mood disorders.

The final search strategy used in OVID (https://ovidsp.ovid.com accessed on 21 October 2021) to search the Medline and Embase biomedical databases was:

(AIM2* or NLRC* or NLRP3* or inflammasome*) AND (monocyte* or lymphocyte* or peripheral* or macrophage* or ASC* or speck*) AND patient* NOT review

The inflammasome portion of the search strategy contained the terms (AIM2* or NLRC* or NLRP3* or inflammasome*) to ensure that all possible assayed inflammasomes were included and avoid missing any data pertinent to the research question. The next portion of the search strategy added terms to include articles about peripheral blood leukocytes and related immune components within these cells. ASC measurements within PBMC were of particular interest due to their clinical accessibility, thus the terms (monocyte*, lymphocyte*, macrophage*, ASC* and speck*) were added. Peripheral* was used to include all possible peripheral blood leukocytes in the search results. Finally, only papers concerning inflammasome activation levels from human peripheral blood leukocyte samples were of interest and thus, an AND patient* term was added to the search strategy.

### 4.2. Development and Validation of a Biological Assay to Detect Activation of the NLRP3 Inflammasome

NLRP3 is expressed by innate immune cells which are accessible in peripheral blood [12]. For quantity-limited material such as primary PBMC, fluorescence microscopy of intact cells is a desirable technical approach which conserves quantity of samples. The hallmark of NLRP3 system activation is recruitment of ASC and pro-caspase-1 with NLRP3 protein, and the release of IL-1 beta and IL-18 [11]. Once ASC is combined with NLRP3, it can be visualized in aggregate as a speck-like protein which can be used as a proxy of activation for the NLRP3 system. Here, we developed an immunofluorescence method to detect the ASC specks in PBMC.

#### 4.2.1. Cell Culture

The NLRP3 inflammasome functions as a stress response; therefore, the experiments for technical validation utilized PBMC from unaffected individuals. Frozen PBMC from healthy donors (ATCC, Manassas, VA, USA, #PCS-800-011) were thawed and plated onto glass coverslips treated with poly-D-lysine (Fisher Scientific, Waltham, MA, #08-774-383) at a density of 2 × 10^6^ cells/mL in RPMI 1640 medium (Life Technologies, Burlington, ON, Canada, #11875093) containing 10% heat-inactivated fetal bovine serum (FBS; Life Technologies, Burlington, ON, Canada, #10082147). The validation experiment in Figure 1A was plated at a density of 1 × 10^6^ cells/mL.

Cells were rested overnight at 37 °C and 5% CO_2_. NLRP3 activation was induced through a defined 2-step pharmacological approach. Cells were challenged with 100 ng/mL ultrapure LPS for 3 h (InvivoGen, San Diego, CA, USA, #tlrl-3pelps) to prime NLRP3 expression. Cell culture medium was removed and replaced with serum-free medium containing either 10 µM nigericin (60 min; Sigma-Aldrich, Saint Louis, MO, USA, #N7143) or 5 mM ATP (30 min, BioShop, Burlington, ON, Canada, #ATP007) to activate the NLRP3 inflammasome. To confirm specificity, we tested the activation response after treating cells with an NLRP3 inhibitor, MCC950 (InvivoGen, San Diego, CA, USA, #inh-mcc) [20]. Cells were treated with 30 min of 0.8 nM–100 nM MCC950 (IC50 = 8 nM) prior to challenge with nigericin or ATP. Following treatment, serum-free supernatant was stored at −80 °C until assayed for IL-1 beta and caspase-1. The remaining cells were prepared for ASC immunofluorescence.

#### 4.2.2. Intracellular ASC Speck Formation

When activated, cytosolic ASC oligomerizes as the inflammasome complex forms, resulting in a single bright ‘speck’ per cell when tagged with immunofluorescent antibodies [37,38]. Specks can be quantified using local intensity thresholds. Immediately following treatments, wells were washed with PBS and fixed for 15 min in 4% paraformaldehyde (PFA, BioShop, Burlington, ON, Canada, #PAR070) at room temperature. Following fixation, wells were simultaneously permeabilized and blocked in 10% goat serum (Life Technologies, Burlington, ON, Canada, #50197Z) with 0.5% Triton X-100 (Sigma-Aldrich, Saint Louis, MO, USA, #X100) for 30 min at 37 °C. This buffer was used as the diluent for all antibodies.

Cells were probed with anti-ASC (1:200; Adipogen, San Diego, CA, USA, #AG-25B-0006) overnight at 4 °C, washed, and then incubated with anti-rabbit Alexa Fluor 568 (1/1000; Life Technologies, Burlington, ON, Canada, #A-11011) for 1 h. Cells were washed with PBS and mounted on glass slides (Fisher Scientific, Waltham, MA, #12-550-15) in Prolong Diamond mounting medium containing DAPI (Life Technologies, Burlington, ON, Canada, #P36971). Slides were visualized at 20X on an epifluorescent microscope (Zeiss, Jena, Germany, Axio Imager M2). The presence of ASC specks was quantified using Volocity 6.0 (Perkin Elmer, Waltham, MA, USA) using the ‘Find 2D Spots’ tool. Results were expressed as the ratio of ASC specks to the total cell count, measured by DAPI staining and quantified using the ‘Find Objects’ tool.

#### 4.2.3. Protein Expression of Caspase-1

Upon activation of the NLRP3 inflammasome, caspase-1 is cleaved into p10 and p20 subunits, and secreted from the cell [37]. Caspase-1 p10 was measured using a standard ELISA procedure in plasma samples and assayed in duplicate. Supernatant samples were incubated in high protein binding plates (Greiner, Monroe, NC, USA, #655061) overnight at 4 °C. Wells were washed with PBS-T (Sigma-Aldrich, St. Louis, MO, USA, #P3563) and blocked in 2% bovine serum albumin (BSA; Sigma-Aldrich, St. Louis, MO, USA, #A9418) for 1 h at room temperature. Samples were probed with anti-caspase-1 p10 (1/1000; Abcam, Waltham, MA, USA, #ab62698) for 1 h, washed, and probed with anti-rabbit-HRP (1/1000; Cell Signaling Technology, Danvers, MA, USA, #7074) for 1 h. After washing, the reaction was developed with TMB solution (Cell Signaling Technology, Danvers, MA, USA, #7004). Then, 1M HCl was used to stop the reaction, and the absorbance was read at 450 nm on a microplate reader (BioTek, Winooski, VT, USA, Synergy H1).

#### 4.2.4. Protein Expression of IL-1 Beta

Protein levels for the pro-inflammatory cytokine IL-1 beta was measured simultaneously in plasma using a high sensitivity human ELISA kit (Millipore, Burlington, MA, USA, #HSTCMAG-28SK). Samples were assayed in duplicate, bound to magnetic beads with specific capture antibodies, and probed with the provided cocktail of biotin-labeled detection antibodies followed by phycoerythrin-conjugated streptavidin. Samples were analyzed on Luminex MAGPIX (Luminex, Austin, TX, USA) with xPONENT 4.2 (Luminex, Austin, TX, USA). Cytokine concentrations were quantified using a 5-parameter logistic curve on Milliplex Analyst 5.1 (Millipore, Burlington, MA, USA).

### 4.3. Assessment of Levels of NLRP3 Inflammasome Activation in Adolescent Patients with Mood Disorders

#### 4.3.1. Patient Recruitment and Eligibility

Patients were recruited by Dr. Kathryn Cullen and Dr. Bonnie Klimes-Dougan at the University of Minnesota. Clinical diagnoses were assigned based on the Kiddie Schedule for Affective Disorders and Schizophrenia for School-Age Children-Present and Lifetime (K-SADS-PL). This project received research ethics approvals at the University of Toronto (RIS Protocol #32212) and IRB approval at the University of Minnesota (study #1507M75201, PI: Dr. Kathryn Cullen).

Subjects were eligible for study entry if they: (1) were aged 13–19 years; (2) had a DSM-IV diagnosis of any mood disorder; and (3) were fluent in English. Exclusionary criteria were: (1) a history of active substance abuse or dependence in the past 3 months, with the exception of nicotine; (2) a history of neurological disease.

#### 4.3.2. Blood Collection and Isolation of Plasma and Peripheral Blood Mononuclear Cells

First, 8 mL of whole blood was collected using EDTA coated interior vacutainer tubes (BD, Franklin Lakes, NJ, USA, #366643) from all participants by Dr. Cullen and Dr. Klimes-Dougan. Whole blood samples were shipped to Dr. Andreazza’s laboratory for processing and analysis. This study has been approved by the Research Ethics Board at the University of Toronto (#32212).

For plasma extraction, the blood was mixed well by inverting the tube 10 times at a 90-degree angle. PBMC were extracted by Ficoll-Paque density-gradient centrifugation. Then, 8 mL of blood was carefully layered on 10 mL of Ficoll-Paque plus (GE Healthcare, Piscataway, NJ, USA, #71-7167-00 AG). Each tube was centrifuged at 400 g for 40 min at room temperature. Plasma was removed from the top layer and aliquoted for storage at −80 °C.

The layer of white blood cells, which appears as a ring, was extracted and transferred to another 15 mL falcon tube containing 10 mL of PBS pH 7.4 and centrifuged at 400 g for 10 min at room temperature. The supernatant was removed, and the pellet was resuspended in 10 mL of PBS and centrifuged again at 400 g for 10 min at room temperature. The washing process was repeated and at the end of the third wash, the pellet was resuspended in 1 mL of freezing media containing RPMI 1640 medium (Life Technologies, Burlington, ON, Canada, #11875093) with 10% dimethylsulfoxide (DMSO; Sigma-Aldrich, St. Louis, MO, USA, #D8418), and aliquoted into 1.5 mL microcentrifuge tubes. White blood cells were slow frozen and kept at −80 °C until assayed.

### 4.4. Statistical Analysis

Statistical analyses were performed using GraphPad 9.0.0 (San Diego, CA, USA) for Windows, with the level of significance set at *p* < 0.05 for all tests. In the assay validation of NLRP3 endpoints, statistical analysis of differences observed between cell treatment groups was performed by one-way ANOVA using a pairwise multiple comparison procedure (Šídák’s multiple comparison test) for correction. Paired *t*-tests were used for comparisons in patient samples between untreated and NLRP3 activated PBMC, as well as measurements from first and second visits.

## Figures and Tables

**Table 1:** Neuropsychiatric Studies Investigating NLRP3 Inflammasome Activation in Mood Disorders. Seven articles [24,25,26,27,28,29,30] investigated NLRP3 inflammasome activation in patients suffering from bipolar disorder and major depressive disorder. All studies use PCR, assay ELISA, or Western blotting to measure mRNA and protein expression in human blood samples containing either PBMC, serum, or plasma.

Reference	Number of Patients			Sample	Assays
	Healthy	BD/MDD	Medicated		
Alcocer-Gómez et al., (2014)	20	20	20	PBMC, Serum	PCR, ELISA, Western Blot
Momeni et al., (2016)	43	38		PBMC	PCR
Alcocer-Gómez et al., (2017)	20	20	194	PBMC, Serum	PCR, ELISA
García-Álvarez et al., (2018)	80	102		PBMC, Plasma	Western Blot
Scaini et al., (2018)	25	31		PBMC	PCR, Western Blot
Taene et al., (2020)	20	20	20	PBMC, Serum	PCR
Li et al., (2021)	24	24		Serum	PCR, ELISA

**Table 2:** Protein and Gene Expression Levels of the NLRP3 Inflammasome in Mood Disorder Studies. No studies measured ASC protein levels. * Denotes one study which demonstrated decreased levels of the NLRP3 inflammasome specifically in MDD patients taking medication [26].

Reference	NLRP3		ASC		Caspase-1		IL-1 beta		IL-18	
	mRNA	Protein	mRNA	Protein	mRNA	Protein	mRNA	Protein	mRNA	Protein
Alcocer-Gómez et al., (2014)	Increase		Increase		Increase			Increase		Increase
Momeni et al., (2016)			Increase							
Alcocer-Gómez et al., (2017)	Increase *							Increase		Increase
García-Álvarez et al., (2018)		Increase								
Scaini et al., (2018)	Increase		Increase		Increase			Increase		Increase
Taene et al., (2020)	Increase				Increase					
Li et al., (2021)	Increase	Increase				Increase	Increase	Increase		

**Table 3:** Patient cohort demographics and medication use. NLRP3 inflammasome testing was conducted in adolescent patients with mood disorders (n = 7), including two adolescents with bipolar spectrum disorders and five adolescents with depressive disorders. Abbreviations: selective-serotonin reuptake inhibitor (SSRI), serotonin-norepinephrine reuptake inhibitor (SNRI).

	Total Mood Disorders		Bipolar Disorders		Depressive Disorders	
		(n = 7)		(n = 2)		(n = 5)
Age, M (SD)		16.9 (1.68)		17.5 (0.71)		16.6 (1.95)
Sex, N (%)						
	Male	2 (29%)		0 (0%)		2 (40%)
	Female	5 (71%)		2 (100%)		3 (60%)
Medication, N (%)						
	SSRI	4 (57%)		0 (0%)		4 (80%)
	SNRI	1 (14%)		1 (50%)		0 (0%)
	None	2 (29%)		1 (50%)		1 (20%)

## Data Availability

The data presented in this study are available on request from the corresponding author. The data are not publicly available due to privacy and ethical restrictions.
